# Superior Keratoconus in Attention-Deficit Hyperactivity Disorder (ADHD): A Case Report and Literature Review

**DOI:** 10.7759/cureus.76013

**Published:** 2024-12-19

**Authors:** Elishai Assayag, David Zadok, Moshe Carmel, Adi Abulafia, Yishay Weill

**Affiliations:** 1 Department of Ophthalmology, Shaare Zedek Medical Center, Faculty of Medicine, Hebrew University of Jerusalem, Jerusalem, ISR

**Keywords:** attention-deficit hyperactivity disorder, corneal tomography, eye rubbing, posterior subcapsular cataract, superior keratoconus

## Abstract

Keratoconus is a progressive corneal ectasia that may lead to severe visual impairment. Superior keratoconus (SK) is an uncommon form of the disease, and few cases have been reported thus far. We present an unusual SK case and a literature review of this rare diagnosis. A 49-year-old man presented to the ophthalmology clinic complaining of decreased vision in his right eye. Previous medical and ocular histories included medically treated attention-deficit hyperactivity disorder (ADHD) and keratoconus, which were diagnosed only in adulthood. A dense posterior subcapsular cataract was observed in the right eye. Corneal tomography revealed bilateral asymmetric SK, which was more severe in the right eye. The patient demonstrated unique eye-rubbing habits, which helped him cope with his ADHD symptoms and correlated with the keratoconus severity in each eye. In a literature review, 11 previously published cases of SK were summarized, none of which were associated with ADHD or included ipsilateral cataracts as in our patient. In conclusion, ADHD may cause repetitive eye rubbing and subsequent uncommon corneal ectatic changes, such as SK. Early recognition and characterization of eye rubbing can be crucial in detecting coexisting mental and ocular disorders.

## Introduction

Keratoconus is a predominantly bilateral, asymmetric ectasia that may cause progressive corneal thinning and steepening, high irregular astigmatism, and severe vision loss [1]. Apart from a positive family history of keratoconus, several systemic and ocular conditions have also been associated with the disease. These include atopic and allergic illnesses, asthma, and eczema [2].

Eye rubbing is another major risk factor for keratoconus, whether the rubbing is due to itchy eyes or other reasons [3]. Several psychiatric disturbances have been linked to repetitive eye rubbing and subsequent development of keratoconus [4]. A large population-based study found that in this context, keratoconus was associated with attention-deficit hyperactivity disorder (ADHD) [5].

Typically, the most protruded area of a keratoconic cornea is located inferior to the horizontal midline, presumably due to the increased vulnerability of epithelial cells in the inferior corneal quadrants [6]. Superior keratoconus (SK) is a rare variant of the disease, comprising less than 1% of all cases [7]. The few reported cases of SK were associated with common risk factors for keratoconus, particularly eye rubbing [7-15]. Psychiatric comorbidities, including ADHD, were not mentioned in any of the cases. Herein, we report an uncommon case of ADHD-associated SK and a literature review of previously published SK cases.

## Case presentation

A 49-year-old man was referred to the preoperative cataract clinic due to blurred vision in his right eye. His medical history was positive for medically treated ADHD, which was suspected in childhood but officially diagnosed only years later. Ocular history included keratoconus in the right eye, diagnosed 15 years prior and until recently was well-managed with spectacle correction alone after several unsuccessful contact lens fitting attempts. The patient denied a family history of keratoconus and ruled out conditions such as atopy, asthma, allergic conjunctivitis, and obstructive sleep apnea. He confirmed repetitively rubbing both eyes since childhood, especially the right eye.

On examination, the best corrected visual acuity (BCVA) was 20/80 with -3.25 -6.00 X 11° and 20/20 with -2.25 -1.25 X 160° in the right and left eye, respectively. External examination unveiled no suggestive findings, such as dermatitis or ptosis. On slit-lamp biomicroscopy, the eyelids and lashes appeared normal. The conjunctiva was quiet, without a papillary reaction in both eyes. No marked protrusion, thinning, scars, striae, or vascularization were noted in both corneas. A dense posterior subcapsular cataract was observed in the superior half of the right lens, and the left lens was clear. Intraocular pressure and posterior segments were normal in both eyes.

**Figure 1:**
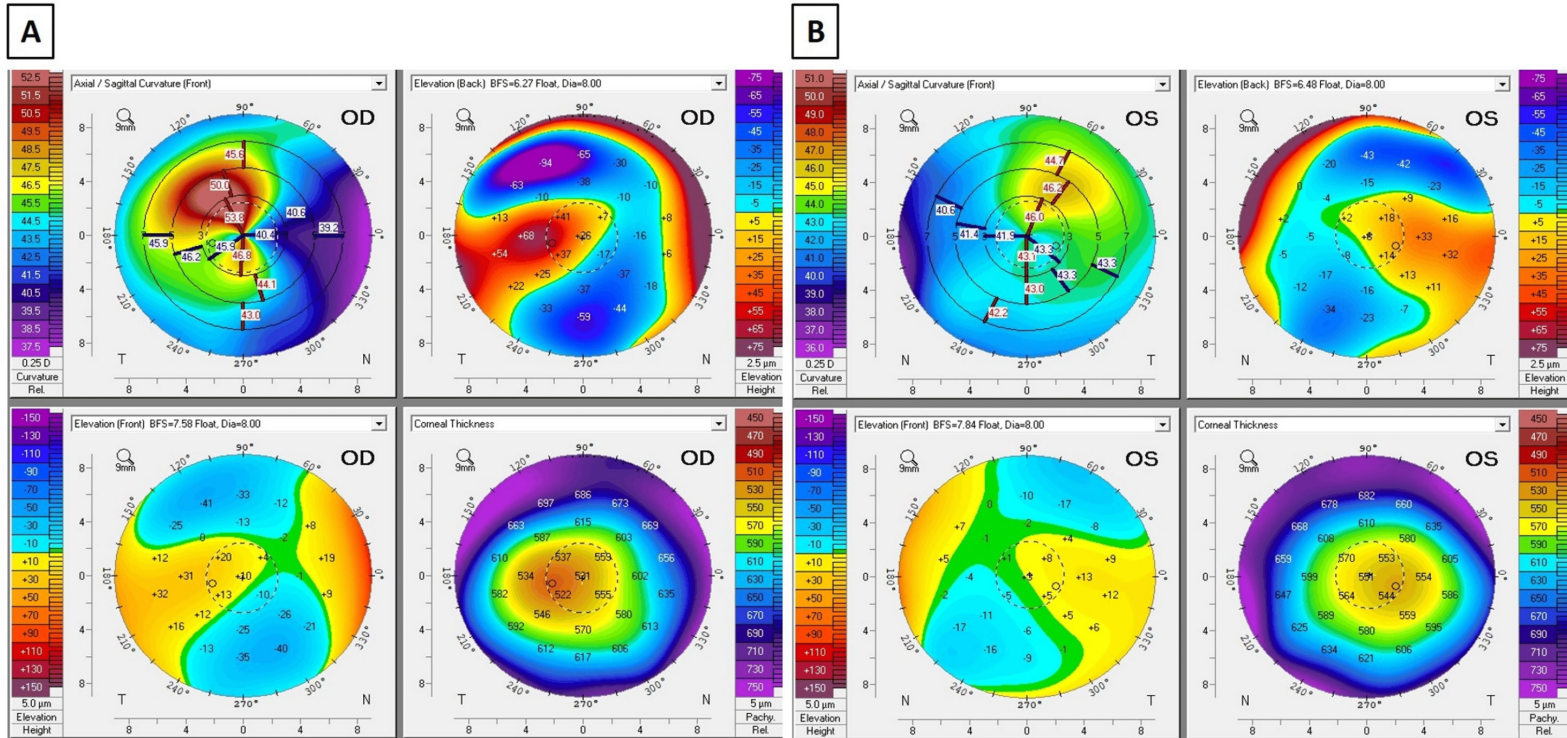
Bilateral superior keratoconus in a 49-year-old patient with ADHD ADHD: attention-deficit hyperactivity disorder Scheimpflug-based tomography (Pentacam, Oculus, Wetzlar, Germany) images of the right (A) and left (B) eye demonstrating bilateral superior keratoconus. The thinnest points are 515 μm and 541 μm in the right and left eye, respectively, and are located approximately on the horizontal midline. Note the relative superior corneal thinning correlating with the locations of the steepest points

Scheimpflug-based tomography (Pentacam, Oculus, Wetzlar, Germany) demonstrated superior steepening and irregular, asymmetric astigmatism in both eyes, more pronounced in the right eye (Figure 1). K-Max values were 53.8D (1.29 mm above the horizontal midline) in the right eye and 46.3D (1.76 mm above the horizontal midline) in the left eye. The thinnest points were 515 μm and 541 μm in the right and left eye, respectively, and were located approximately on the horizontal midline. Maximal posterior elevation was 68 μm and 33 μm, and maximal anterior elevation was 33 μm and 14 μm in the right and left eye, respectively. All four maximal elevation points were measured on or slightly superior to the horizontal midline.

In repeated anamnesis, the patient stated that since an early school age, and to a much lesser extent since his ADHD is treated, he regularly rubs his eyes to cope with stress and improve concentration during learning and working tasks. He demonstrated a rubbing routine during which he strongly squeezes his thumb or the back tip of a pencil against the upper eyelid and applies pressure on the globe, far more frequently in the right eye. A diagnosis of SK was made, and the patient was referred for further consultations at our cornea and cataract clinics. 

## Discussion

SK is an uncommon form of the disease, and only a few cases have been published thus far [7-15]. We conducted a literature review which yielded 11 previously published SK cases (Table 1). Most patients presented between the second and fourth decades of life and were diagnosed with bilateral, asymmetric SK. Habitual eye rubbing was documented in 7/11 cases. The severity of SK at presentation was significantly variable. Two eyes recovered after ptosis correction, four were successfully managed with rigid gas permeable (RGP) contact lenses only, two presented after corneal hydrops in one eye, four underwent corneal cross-linking, and two required keratoplasties. Keratoconus progression after the initial management was not documented.

**Table 1:** Previously reported cases of superior keratoconus BCVA: best corrected visual acuity; KCN: keratoconus; RGP: rigid gas permeable; N/A: not available; ATR: against the rule; PMD: pellucid marginal degeneration; CF: counting fingers; CXL: corneal cross-linking; PK: penetrating keratoplasty; ALK: anterior lamellar keratoplasty; OD: right eye; OS: left eye; OU: both eyes

Authors (year)	Number of patients	Age (y)/gender	Associated conditions	Laterality/symmetry	Eye rubbing	BCVA	KCN signs on slit lamp	Main refractive/topographic/ tomographic findings	Management and course
Eiferman et al. (1993) [8]	1	35/F	N/A	Unilateral	N/A	Worse than 20/20 OS	-	Superior corneal ectasia, irregular astigmatism	BCVA improved to 20/20 with RGP contact lens
Prisant et al. (1997) [9]	1	57/M	-	Bilateral/asymmetric	No	20/25 OD 20/400 OS	OS	OD: Superior steepening, maximal corneal power 53.7 D OS: N/A	N/A
Kim et al. (2000) [10]	1	62/M	Ptosis OU	Bilateral/symmetric	Yes	20/20 OU	-	Manifest refraction: +2.00 -1.50 X 90 OU, Superior steepening OU	All signs and symptoms resolved 3 months after blepharoplasty, and visual acuity was 20/20 OU without correction
Weed et al. (2005) [7]	2	15/M	-	Unilateral	No	20/20 OU	OS	OS: Superior steepening, Sim K readings: 59.03/52.26 X 18°	Successful adaptation to RGP contact lenses OU
		12/M	Asthma, Eczema, hay fever	Unilateral	Yes	20/20 OU	OD	OD: Superior steepening, Sim K readings: 52.95/44.45 X 22°	Successful adaptation to RGP contact lens OD
Chiang et al. (2007) [11]	1	30/M	N/A	Bilateral/symmetric	N/A	20/40 OD 20/100 OS	OU	OU: Superior steepening, ATR astigmatism with asymmetric bow-tie configuration, long distance between cone apex and thinnest point, PMD signs	N/A
Tananuvat et al. (2009) [12]	1	32/M	-	Bilateral/asymmetric	Yes	20/1200 OD 20/20 OS	OD	Keratometry readings: OD: N/A, OS: 40.0/47.0 X 130° tomography N/A OD, superior steepening OS	Presented with corneal hydrops and edema OD and treated with lubricants and hypertonic saline. Echocardiography revealed a myxomatous mitral valve
Rogers and Attenborough (2014) [13]	2	26/F	Asthma	Bilateral/asymmetric	Yes	20/60 OD CF 3m OS	OU	Marked superior steepening and thinning OU	Treated for corneal hydrops OS 3 years prior. Referred to CXL OD and PK OS
		18/F	-	Bilateral/symmetric	Yes	20/50 OU	OU	Superior steepening and irregular asymmetric astigmatism OU	Underwent a trial of rigid gas-permeable contact lenses fitting
Galperín and Berra (2021) [15]	1	16/M	-	Bilateral/asymmetric	Yes	CF 2m OD 20/30 OS	OD	Severe superior steepening and thinning OD	Referred to ALK OD and CXL OS
Mounir and Mostafa (2021) [14]	1	26/M	-	Bilateral/asymmetric	Yes	20/50 OD 20/70 OS	OU	Superior steepening and thinning OU	Underwent CXL OU. Improvement in BCVA and keratometry in a 3-year follow-up

SK may even be more uncommon than presumed, as some cases were diagnosed utilizing old corneal imaging technologies [8]. It is possible that the superior steepening and irregular astigmatism in some cases resulted from other conditions, such as warpage, and that modern tomography would have provided the necessary data to confirm or rule out the diagnosis. In one case, the topographic and refractive findings completely resolved after ptosis correction, making a diagnosis of SK less probable [10].

In the history-taking stage of keratoconus diagnosis, practitioners may automatically associate repetitive eye rubbing with itchy eyes due to allergic conjunctivitis, dry eyes, or atopy. However, ocular surface irritation is not always the culprit for eye rubbing. Habitual eye rubbing can result from psychogenic factors such as emotional stress, psychosis, and compulsion [4]. Accordingly, eye rubbing-induced bradycardia, a result of intentional activation of the oculocardiac reflex in order to relax, can partially explain the higher rates of keratoconus among patients with ADHD [5].

Our case of SK is unique for several reasons. Firstly, most SK case reports did not elaborate on the exact eye-rubbing routines. Only the two patients described by Rogers and Attenborough demonstrated their eye-rubbing pattern, in which digital pressure was applied against the upper globe in an extremely similar manner to our patient's habit [13]. We suggest that this form of rub may put patients at risk for SK, emphasizing the importance of acquiring an exact description of the rubbing method.

Secondly, to our knowledge, this is the first documented ADHD-associated case of SK. Apart from our patient's need for self-relaxation when attempting to concentrate, his history and examination did not reveal other factors that provoke chronic eye rubbing, as in other cases linking ADHD to keratoconus [16]. Furthermore, five patients with SK described repetitive eye rubbing despite no apparent predisposing conditions [12-15]. This stresses the importance of thorough history-taking and perhaps even a multidisciplinary approach in characterizing eye rubbing.

Lastly, our patient presented at a relatively young age with a unilateral cataract in the right eye, which was rubbed more vigorously and exhibited a more severe SK. A cataract was not seen in any of the SK cases we reviewed, including those who presented at an older age. In a study on atopic dermatitis patients, eye rubbing was significantly associated with cataract progression, and the common type of cataract was the anterior and posterior subcapsular [17], as in our patient. We find it reasonable to assume that the unique, long-standing, asymmetric form of eye rubbing contributed to the development of clinically significant cataracts and a more severe SK in the same eye. Moreover, the superior lens quadrants in which the cataract was denser correlated in location with the ectatic superior cornea, perhaps highlighting eye rubbing as a common offending mechanism.

## Conclusions

This rare case of ADHD-associated SK underscores the importance of detailed characterization of eye-rubbing habits and its probable role in the pathogenesis of keratoconus. Practitioners from different disciplines may be required to collaborate to comprehend why a specific rubbing pattern manifests and the potential consequent ocular damage. Pediatricians, neurologists, and ophthalmologists should be cognizant of this rare but possible presentation of ADHD-related keratoconus.
